# Integrating Cultural Humility Into Infant Safe Sleep Counseling: A Pediatric Resident Simulation

**DOI:** 10.7759/cureus.20847

**Published:** 2021-12-31

**Authors:** Chelsea Moore, Shaina M Hecht, Htayni Sui, Lisa Mayer, Emily K Scott, Bobbi Byrne, Megan S McHenry

**Affiliations:** 1 Department of Pediatrics, Indiana University - Purdue University Indianapolis, Indianapolis, USA; 2 Department of Pediatrics, Indiana University School of Medicine, Indianapolis, USA

**Keywords:** cultural humility, safe sleep, pediatrics education, simulation in medical education, global health education

## Abstract

Introduction: Co-sleeping with infants is a common practice across cultures, but pediatricians may struggle to engage in patient-centered conversations about infant sleep practices with non-native English- speaking families. Cultural humility is a critical skill to utilize when engaging in cross-cultural conversations. We designed a simulation for pediatric residents to counsel on safe sleep and enhance skills in self-perceived cultural humility and preparedness when caring for diverse patient populations.

Methods: We created a simulation for the second year and senior pediatric residents at a large academic institution focused on a co-sleeping parent and infant from the Burmese community. The Multidimensional Cultural Humility Scale (MCHS) was administered prior to and after the simulation. We also included additional questions regarding changes in knowledge and preparation in engaging in co-sleeping conversations across cultures.

Results: Fifty-seven residents participated. Overall, the mean score of the MCHS significantly increased after the simulation, indicating an increase in self-perceived cultural humility. All participants felt more prepared to have conversations about co-sleeping and to engage in difficult conversations with diverse patient populations, and all learned valuable skills to improve care for future patients. Comments regarding the scenario noted an appreciation for learning more about the Burmese population and understanding new approaches to safe sleep counseling.

Discussion: After this simulated scenario, residents reported increased self-perceived cultural humility, preparedness in counseling on co-sleeping, and skills to engage in difficult conversations with diverse patient populations. Topics such as cultural humility can be incorporated into simulation-based medical education to help improve the care of diverse patient populations.

## Introduction

Co-sleeping is a common practice globally. Considered to be a form of affection, co-sleeping, also known as bed-sharing, is common in many cultures worldwide, with one Canadian study reporting 57% of mothers born in Asia practicing this tradition [1]. Furthermore, recent studies note that the practice of co-sleeping in the United States has increased from 6.5% in 1993 to 24.4% in 2015 [2,3]. Despite the prevalence of co-sleeping, there are several unintentional consequences, including sudden infant death syndrome (SIDS) and accidental suffocation [4-6]. Due to these risks, organizations such as the American Academy of Pediatrics (AAP) support sharing a room with an infant while advising against co-sleeping [7]. Pediatricians can face challenges in safe sleep counseling due to conflicting opinions between the physician and family. These challenges may increase when caring for patients for whom English is not their native language due to a lack of cultural understanding, language barriers, or conflicting opinions [8]. These difficult conversations about safe sleep can lead to the inability of the physician to develop rapport with families and to the distrust of medical advice by patients.

Cultural humility is a valuable skill for all physicians to utilize in patient care. The National Institutes of Health defines cultural humility as "a lifelong process of self-reflection and self-critique whereby the individual not only learns about another's culture, but starts with an examination of her/his own beliefs and cultural identities" [9]. Cultural humility can be mutually beneficial, leading to empowerment, optimal care, respect, and lifelong learning [10]. Developing skills in cultural humility can help physicians empathize with and openly discuss the beliefs and values of patients from different cultures. 

While others have created simulation sessions to improve skills in cultural humility, no studies looking to build skills in safe sleep counseling using cultural humility were found in the literature [11]. We believe cultural humility benefits all patient encounters and is particularly useful in encounters involving co-sleeping. To ensure residents are receiving appropriate training and building the skills necessary for safe sleep conversations, we designed a co-sleeping simulation scenario.

This report presents a simulation focused on providing pediatric residents with the skills to counsel on safe sleep across cultures and reflect on cultural humility. Strategies to mitigate the risks of co-sleeping were also explored in this simulation scenario. This simulation was previously presented as an oral presentation at the Indiana University School of Medicine Education Day and presented virtually in April 2021.

## Materials and methods

This simulation was performed in the simulation laboratory at the Indiana University School of Medicine Simulation Center as part of a mandatory residency simulation curriculum provided annually over five hours of protected educational time. Second-year pediatrics residents and senior residents from multiple pediatric programs, including categorical pediatrics, internal medicine/pediatrics, emergency medicine/pediatrics, pediatrics/psychiatry/child and adolescent psychiatry, and neurodevelopmental disabilities, participated in the simulation. Senior residents refer to those who are in the final year of their pediatrics residency or pediatrics portion of their residency. For this scenario, the simulation was performed in a specialized simulation lab room. From clinical experiences, prior research, and discussions with these families, co-sleeping (which is also known as bed-sharing) was identified as a common practice among the Burmese community and was identified by Burmese families as an area of disagreement with their physicians. This simulation scenario was developed in partnership with a local Burmese refugee organization, a medical director of a large newborn unit, and an experienced simulation instructor. The simulation was approved by the Institutional Review Board of Indiana University. 

Prior to the scenario, the residents were prompted to complete a pre-simulation survey (Appendix A). The survey consisted of 15 questions from the Multidimensional Cultural Humility Scale (MCHS) [12]. The questions were derived from five components of Foronda et al.: cultural humility, including questions about openness, self-awareness, ego-less, supportive interactions, and self-reflection and critique [10]. The responses were measured using a six-point Likert scale (1= strongly disagree, 3=slightly disagree, and 6=strongly agree). A higher score indicated a higher level of cultural humility, except for three items about supportive interactions that were reverse-coded. Immediately after the simulation, the same survey with three additional questions relating to knowledge and preparedness for co-sleeping conversations with different cultures was distributed to all residents for completion (Appendix B). A paired t-test was used to compare the pre- and post-survey responses. P-values of <0.05 were considered significant.

We recommend the following equipment for successful implementation of this simulation case: a chair for the standardized patient (SP), earpiece headphones for the SP, a low-fidelity infant mannequin, an exam table, a chair for the provider, and one-way glass mirror or video recording capabilities. In each simulation, the learners were pediatric residents, pre-assigned to groups consisting of three to five residents per group. The group selected one resident (by group consensus or volunteer) to enter the simulation room while the facilitator brought the remaining residents behind a one-way mirror to watch and listen to the scenario. The SP was an actress hired for the simulation, recruited from a local Burmese community organization. To create the most accurate scenario, the SP underwent a two-hour training where she learned about the simulation, how to react to different questions asked by the residents, and insights into the resident’s possible thought process. The facilitator of the simulation was a pediatrician and faculty member. When necessary, the facilitator helped to guide the SP in answering questions from the resident via the earpiece headphone.

Simulations for second-year and senior residents were administered on two separate days and were one of eight scenarios that the residents completed. The simulation was performed in a mock hospital room at the simulation center. Each scenario ran for approximately 10 minutes, with a 15-minute debriefing of the simulation afterward. Prior to entering the simulation room, the resident participating in the scenario was told that their patient was a healthy four-week-old Burmese male infant coming in for their first well-child check (Appendix C). As part of the standard infant clinical visit, residents would either ask about the infant’s sleeping situation or the facilitator would prompt the SP to bring up her worries about the death of a child in her community due to co-sleeping. The SP was coached to give specific clues to guide the resident into a discussion about sleep practices. 

The residents who were not participating in the scenario were encouraged to take notes and complete an evaluation (Appendix D). The evaluation forms guided the observing residents to focus on the important parts of the encounter between the resident and the SP but were not included in data collection or analysis to avoid leniency bias when scoring their peer. After the simulation, residents were given 15 minutes for a debriefing discussion about the scenario, in which they also received feedback. The debriefing session was a semi-structured discussion where the SP shared her perspectives and the residents shared their thoughts after the session. Mitigation strategies to reduce the risks of co-sleeping were discussed with the residents, which included never leaving the infant alone on the bed, ridding the bed of pillows, blankets, or any other soft surfaces, lowering the bed onto the floor and away from any walls, only one parent bedsharing with the infant, placing the infant inside a crate or box in the bed, and reducing alcohol and tobacco use when bedsharing [13]. The residents were provided verbal and written information on safe sleep methods and tips on how to confidently approach co-sleeping conversations, such as keeping the conversation free of judgment and asking open-ended questions. The Indiana Department of Health created a resource on safe sleep that was provided to residents after the simulation [14]. Additionally, the SP was able to give feedback on how the residents approached the safe sleep conversation and how they might be able to improve their discussions with future Burmese patients. A post-survey (Appendix B) was distributed to all residents after debriefing was completed.

## Results

Fifty-seven residents participated in this simulation over the course of two days in September and November 2020. The learners were from four different pediatric residency programs within a single institution (categorical pediatrics, internal medicine/pediatrics, emergency medicine/pediatrics, and neurodevelopmental disabilities). With regard to training status, there were 27 second-year residents and 30 senior residents. A summary of participant characteristics is shown in Table 1.

**Table 1:** Characteristics of 57 residents participating in the simulation No.: number, PGY: post-graduate year ^a^Refers to PGY-3, or 4 and 5 if resident in a program other than categorical pediatrics residency.

Characteristic	No. (%), N=57
Gender	
Male	20 (35)
Female	37 (65)
PGY status	
2	27 (47)
3^a^	30 (53)
Program	
Categorical pediatrics	39 (68)
Internal medicine/pediatrics	15 (26)
Emergency medicine/pediatrics	1 (2)
Pediatrics/psychiatry/child and adult psychiatry	0 (0)
Neurodevelopmental disabilities	2 (4)

During the simulation, all participants were able to identify the issue of co-sleeping and engaged in an appropriate conversation about co-sleeping with the SP. Fifty residents completed both the pre- and post-simulation surveys, and seven residents only completed the post-session survey. The average MCHS score for all residents improved significantly after the simulation (mean: 67.3 vs. 71.2, p-value <0.001). Second-year residents had the largest change in average MCHS scores, increasing from 67.98 to 73.56. The increase in average MCHS score was less for the senior residents as compared to the second-year residents but was still statistically significant (65.74 to 68.68, p-value 0.013). Regarding each of the five components of cultural humility, the overall average difference between Likert scores for openness, self-awareness, egoless, supportive interactions, and self-reflection and critique were +5.21, +4.61, +4.35, +4.20, and +5.30, respectively. 

At the end of the simulation, participants reported feeling more confident about navigating co-sleeping conversations in the future. All participants noted feeling better prepared to have a conversation about co-sleeping (mean Likert score: 5.35, SD: 0.72), feeling better prepared to engage in difficult conversations with a diverse patient population (mean: 5.09, SD: 0.68), and all participants felt as if they learned valuable skills to improve the care of future patients because of this scenario (mean: 5.15, SD: 0.69). There were no participants who disagreed with these three statements. Table 2 shows the results of the additional questions.

**Table 2:** Results of the additional post-simulation survey questions (N=57) No.: number

	No. (%), N=57						
Item	Strongly disagree	Moderately disagree	Slightly disagree	Slightly agree	Moderately agree	Strongly agree	No response
I feel better prepared to have a conversation about co-sleeping because of this scenario.	0 (0)	0 (0)	0 (0)	6 (10)	22 (39)	28 (49)	1 (2)
I feel better prepared to engage in difficult conversations with a diverse patient population because of this scenario.	0 (0)	0 (0)	0 (0)	7 (12)	33 (58)	15 (26)	2 (4)
I learned valuable skills to improve care of my future patients because of this scenario.	0 (0)	0 (0)	0 (0)	8 (14)	30 (53)	18 (32)	1 (2)

Most residents had not received previous training on risk mitigation for co-sleeping, but nearly all reported verbally during the debriefing that they would like to receive training on this topic. Within the free-text comments of the post-simulation survey, many residents noted that the simulation was culturally and practically beneficial. Comments included:

"[This simulation was] excellent and instructive. It was very helpful to see it in a practice scenario."

"It was nice to hear and learn about approaches to these issues."

"I enjoyed learning more about cultural practices within the Burmese population, especially regarding bonding and co-sleeping."

"[The simulation was] a very important station."

## Discussion

This simulation session was created for pediatric residents to experience the complexities of a conversation about co-sleeping. The session focused on providing skills to navigate the co-sleeping conversation, offered mitigation strategies for safer co-sleeping, and most importantly, helped the residents explore their self-awareness of cultural humility. The strength of this scenario is that we developed it with input from members of our local Burmese Christian community. However, we believe that it would easily be adapted to other immigrant and refugee populations or for non-English-speaking patients. After this simulation, pediatric residents gained experience navigating conversations about co-sleeping in a controlled simulation setting and were able to reflect on their cultural humility.

Overall, results showed that the simulation led to an increase in all areas of cultural humility among the 57 residents who participated in or observed the scenario. Both second-year and senior residents significantly improved their scores. The residents also reported gaining valuable skills to improve the care of their future patients, with all scoring that they felt better prepared to engage in a co-sleeping conversation and to have difficult conversations with diverse populations.

Residents are trained to counsel on safe sleep using the AAP’s recommendations, including placing the infant on their back for every sleep, using a firm sleep surface, keeping soft objects and loose bedding away from the infant, and room-sharing without bed-sharing [7]. While the goal of the simulation was to promote safe sleep, during debriefing, most residents reported that they had not been exposed to education on risk mitigation for co-sleeping. While it would be optimal for all parents to follow all the recommendations put forth by the AAP, it is also essential to consider the family’s beliefs and environment when engaging in shared decision-making. In situations where strict adherence to recommendations is not feasible, either practically or culturally, risk mitigation will often enable providers to work alongside the family to create the safest environment possible. For safe sleep counseling, risk mitigation strategies include never leaving the infant alone on the bed, ridding the bed of pillows, blankets, or any other soft surfaces, lowering the bed onto the floor and away from any walls, only having one parent bedsharing with the infant, placing the infant inside a crate or box in the bed, and reducing alcohol and tobacco use when bedsharing [13]. More education surrounding the idea of risk mitigation, especially for safe sleep counseling, should be addressed elsewhere in the medical education of physicians. For example, a recent published module trains medical students on risk mitigation strategies regarding opioid use [15].

A unique aspect of the development of this simulation was our partnership with the local Burmese refugee population. By learning more about their community’s experiences with physicians, we strived to create a realistic conversation about co-sleeping. The inclusion of a trained Burmese actress also added to the authenticity of the scenario and enhanced the debriefing feedback. By collaborating with local immigrant and refugee organizations, we learned about the challenges that people face when interacting with healthcare providers. Community-engaged research prioritizes issues of concern for the local community and has the potential to alleviate health inequities faced by these populations. We believe that medical simulations are enhanced by incorporating elements of local community needs, social determinants of health, and cultural humility. To better serve the community and promote diversity and inclusion education, we encourage reaching out to minority groups and populations within an area when developing resident simulation scenarios.

One limitation to our project is the lack of data regarding the resident’s level of comfort with co-sleeping conversations prior to the scenario. Due to concerns about overconfidence bias, we did not evaluate their comfort on this topic before the scenario. For future simulations, we recommend incorporating a retrospective pre- and post- survey design to avoid overconfidence bias while still gaining insight on participant comfort and experience. Additionally, due to time restraints, only one resident per group participated in the simulation, and the rest of the group were observers. This may have limited the learning opportunity for the observing residents, though emerging literature shows that observation-based simulation can still improve knowledge [16]. However, it is possible that this limitation affected our results or the direction of the debriefing conversation, as this volunteer resident may have been the most confident in their clinical skills of the group. In the future, we would consider randomly assigning the residents to participate in the simulation to avoid potentially biasing our results. Finally, we recognize that health practices may vary among and within cultural groups.

## Conclusions

In conclusion, this simulation provided residents with the skills to adequately lead a co-sleeping conversation with a mother from a minority community and better self-awareness of their cultural humility. By developing this simulation with input from a local refugee community as well as the inclusion of a Burmese SP, we provided an authentic learning experience for the residents. With the addition of a discussion of risk mitigation strategies, the simulation gave pediatric residents more tools to help mothers choose the safest sleep possible for their infants. Cultural humility and knowledge of risk mitigation strategies are essential in leading safe sleep conversations and may improve a patient’s healthcare experience.

## Appendices

Appendix A: Pre-simulation survey

Name: ______________________________________      

Please mark the response that describes your stance on the following statements.

**Table 3:** Pre-simulation survey

		1	2	3	4	5	6
1	I am comfortable asking my [patients] about their cultural experience.	Strongly Disagree	Moderately Disagree	Slightly Disagree	Slightly Agree	Moderately Agree	Strongly Agree
2	I seek to learn more about my [patients’] cultural background.	Strongly Disagree	Moderately Disagree	Slightly Disagree	Slightly Agree	Moderately Agree	Strongly Agree
3	I believe that learning about my [patients’] cultural background will allow me to better help my [patients].	Strongly Disagree	Moderately Disagree	Slightly Disagree	Slightly Agree	Moderately Agree	Strongly Agree
4	I seek feedback from my supervisors when working with diverse [patients].	Strongly Disagree	Moderately Disagree	Slightly Disagree	Slightly Agree	Moderately Agree	Strongly Agree
5	I incorporate feedback I receive from colleagues and supervisors when I am faced with problems regarding cultural interactions with [patients].	Strongly Disagree	Moderately Disagree	Slightly Disagree	Slightly Agree	Moderately Agree	Strongly Agree
6	I am known by colleagues to seek consultation when working with diverse [patients].	Strongly Disagree	Moderately Disagree	Slightly Disagree	Slightly Agree	Moderately Agree	Strongly Agree
7	I ask my [patients] about their cultural perspective on topics discussed in session.	Strongly Disagree	Moderately Disagree	Slightly Disagree	Slightly Agree	Moderately Agree	Strongly Agree
8	I ask my [patients] to describe the problem based on their cultural background.	Strongly Disagree	Moderately Disagree	Slightly Disagree	Slightly Agree	Moderately Agree	Strongly Agree
9	I ask my [patients] how they cope with problems in their culture.	Strongly Disagree	Moderately Disagree	Slightly Disagree	Slightly Agree	Moderately Agree	Strongly Agree
10	I wait for others to ask about my biases for me to discuss them.	Strongly Disagree	Moderately Disagree	Slightly Disagree	Slightly Agree	Moderately Agree	Strongly Agree
11	I do not necessarily need to resolve cultural conflicts with my [patient] in [clinical care].	Strongly Disagree	Moderately Disagree	Slightly Disagree	Slightly Agree	Moderately Agree	Strongly Agree
12	I believe the resolution of cultural conflict in [clinical care] is the [patients’] responsibility.	Strongly Disagree	Moderately Disagree	Slightly Disagree	Slightly Agree	Moderately Agree	Strongly Agree
13	I enjoy learning from my weaknesses.	Strongly Disagree	Moderately Disagree	Slightly Disagree	Slightly Agree	Moderately Agree	Strongly Agree
14	I value feedback that improves my clinical skills.	Strongly Disagree	Moderately Disagree	Slightly Disagree	Slightly Agree	Moderately Agree	Strongly Agree
15	I evaluate my biases.	Strongly Disagree	Moderately Disagree	Slightly Disagree	Slightly Agree	Moderately Agree	Strongly Agree

Appendix B: Post-simulation survey

Name:                                                                              Role: (circle one)              Clinician   OR   active observer

Please reflect on this simulation experience. Please mark the response that best describes your stance on the following statements.

**Table 4:** Post-simulation survey

		1	2	3	4	5	6
1	I am comfortable asking my [patients] about their cultural experience.	Strongly Disagree	Moderately Disagree	Slightly Disagree	Slightly Agree	Moderately Agree	Strongly Agree
2	I seek to learn more about my [patients’] cultural background.	Strongly Disagree	Moderately Disagree	Slightly Disagree	Slightly Agree	Moderately Agree	Strongly Agree
3	I believe that learning about my [patients’] cultural background will allow me to better help my [patients].	Strongly Disagree	Moderately Disagree	Slightly Disagree	Slightly Agree	Moderately Agree	Strongly Agree
4	I seek feedback from my supervisors when working with diverse [patients].	Strongly Disagree	Moderately Disagree	Slightly Disagree	Slightly Agree	Moderately Agree	Strongly Agree
5	I incorporate feedback I receive from colleagues and supervisors when I am faced with problems regarding cultural interactions with [patients].	Strongly Disagree	Moderately Disagree	Slightly Disagree	Slightly Agree	Moderately Agree	Strongly Agree
6	I am known by colleagues to seek consultation when working with diverse [patients].	Strongly Disagree	Moderately Disagree	Slightly Disagree	Slightly Agree	Moderately Agree	Strongly Agree
7	I ask my [patients] about their cultural perspective on topics discussed in session.	Strongly Disagree	Moderately Disagree	Slightly Disagree	Slightly Agree	Moderately Agree	Strongly Agree
8	I ask my [patients] to describe the problem based on their cultural background.	Strongly Disagree	Moderately Disagree	Slightly Disagree	Slightly Agree	Moderately Agree	Strongly Agree
9	I ask my [patients] how they cope with problems in their culture.	Strongly Disagree	Moderately Disagree	Slightly Disagree	Slightly Agree	Moderately Agree	Strongly Agree
10	I wait for others to ask about my biases for me to discuss them.	Strongly Disagree	Moderately Disagree	Slightly Disagree	Slightly Agree	Moderately Agree	Strongly Agree
11	I do not necessarily need to resolve cultural conflicts with my [patient] in [clinical care].	Strongly Disagree	Moderately Disagree	Slightly Disagree	Slightly Agree	Moderately Agree	Strongly Agree
12	I believe the resolution of cultural conflict in [clinical care] is the [patients’] responsibility.	Strongly Disagree	Moderately Disagree	Slightly Disagree	Slightly Agree	Moderately Agree	Strongly Agree
13	I enjoy learning from my weaknesses.	Strongly Disagree	Moderately Disagree	Slightly Disagree	Slightly Agree	Moderately Agree	Strongly Agree
14	I value feedback that improves my clinical skills.	Strongly Disagree	Moderately Disagree	Slightly Disagree	Slightly Agree	Moderately Agree	Strongly Agree
15	I evaluate my biases.	Strongly Disagree	Moderately Disagree	Slightly Disagree	Slightly Agree	Moderately Agree	Strongly Agree
16	I feel better prepared to have a conversation about co-sleeping because of this scenario.	Strongly Disagree	Moderately Disagree	Slightly Disagree	Slightly Agree	Moderately Agree	Strongly Agree
17	I feel better prepared to engage in difficult conversations with a diverse patient population because of this scenario.	Strongly Disagree	Moderately Disagree	Slightly Disagree	Slightly Agree	Moderately Agree	Strongly Agree
18	I learned valuable skills to improve care of my future patients because of this scenario.	Strongly Disagree	Moderately Disagree	Slightly Disagree	Slightly Agree	Moderately Agree	Strongly Agree

Appendix C: Simulation case template

Patient age: Four-week old

Chief complaint: Well child check

Physical setting: Outpatient clinic

Brief Narrative Description of Case

A four-week-old Burmese infant presents with his mother for a well child check, where it is discovered that the mother is co-sleeping with her infant. The goal of this simulation case is for pediatric residents to practice counseling on safe sleep while enhancing skills in self-perceived cultural humility and preparedness to care for diverse patient populations.

Primary Learning Objectives

1. Demonstrate skills necessary to care for individuals whose primary language is not English

2. Review knowledge of safe sleep recommendations

3. Explore self-awareness of cultural humility

Critical Actions

The goal is to have the resident ask about the sleeping situation for the infant and provide respectful safe sleep counseling. The resident should at some point ask a question about where the baby sleeps and the SP will describe the “Infant Sleep Back Story” (described below, a story about an infant in her community dying in bed with their parents). The resident should engage the SP about co-sleeping and try to understand her perspective, and if done correctly, the SP may be willing to implement strategies to reduce risks of co-sleeping.

Report Learner Receives Prior to Simulation

Four-week-old Burmese male infant coming for their first well-child check today. Mother speaks English and said an interpreter was not needed. Both the MA and medical student confirm that no interpreter is needed. The trusted and thorough medical student saw the patient first, notes that his birth weight was 3.1 kg, and he’s now 3.94 kg (weight gain 30 g/day since birth), making adequate wet diapers, physical examination completely unremarkable, normal red-light reflex, normal heart rate, no murmurs, normal breathing, no rashes, moving all extremities freely and equally. No concerns. Medical student provided anticipatory guidance on car seat safety and on reasons to call the doctor (temperature of over 100F, decreased wet diapers and feedings).

Initial Presentation

Initial vital signs: BP - 80/40, HR - 110, RR - 40, SpO_2 _- 99, Temp: 97 °C

Overall setting and appearance: Outpatient clinic setting, young Burmese mother sitting with her four-week-old son in a chair next to the exam table

Standardized participants (backstory for Burmese actor/actress): You’ve lived in the United States and (insert your specific local community information as applicable) for the last four years. The goal is to have the resident ask you about the sleeping situation for your infant and provide respectful safe sleep counseling.

HPI: If asked, you nurse your baby more than six times every day, until the baby is finished eating. The baby will also feed overnight. You are unsure of the exact number of feedings because you are sometimes asleep. Makes 8-10 wet diapers a day. Your baby has not been sick or had any fevers/rashes. If asked, no COVID-19 symptoms or known exposures (this may be removed post-COVID-19 pandemic).

Past medical/surgical history: Full-term infant, no surgical history

Medications: Vitamin D

Allergies: NKDA

Social/family history: You learned English in Thailand, while awaiting resettlement with your family. You have a husband and no other children, but you also live with your parents and four younger siblings in a two-bedroom home. You and your husband sleep with your baby on a mattress in a bedframe against the wall. You have two to three blankets and pillows on the bed. You do not smoke or drink. Occasionally one of your younger siblings will come and sleep in your room. Your husband works outside the home, and you used to take college classes but plan to stay home with the baby during this time. You do not currently worry about money or getting enough food. No one in your family, including you, smokes, drinks, or does drugs of any kind.

Physical examination: Normal physical exam findings

**Table 5:** Instructor notes - changes and case branch points

Intervention/time point	SP script	Case progression
Introduction (0-2 minutes)	Answer questions asked by resident	Warm engagement. Looks directly at mother. Smiles. Introduces self. Assesses if they feel comfortable without an interpreter
SP concerns (2-6 minutes)	Introduction of community account of infant death	Support mother in this sad event. Resident asking about what the mother thinks of this situation, what the community thinks.
Safe Sleep Counseling (6-9 minutes)	Discussion of mother’s sleep practices	Probes about specifics regarding sleep situation (number of people in bed, other objects in bed, sleep quality)
End of simulation (10 minutes)	End scenario	Transition to another topic

Ideal Scenario Flow

The goal is to have the resident ask you (the SP) about the sleeping situation for your infant and provide respectful safe sleep counseling.

There are a few ways this could come up:

If asked how often the baby eats every day, say that you breastfeed him six times during the day and a few times at night. You’re unsure of the exact number because you sometimes are asleep. Allow for them to ask a question about where the baby sleeps. Then go into the “Infant Sleeping Back Story.”

If asked how YOU (as a mother) are doing, initially say you are okay. If they ask any additional questions about how you’re doing, say that you have been feeling worried. Then go into the “Infant Sleeping Back Story” below.

If asked where the baby sleeps, say that he sleeps with you, but you are worried. Then go into the “Infant Sleeping Back Story” below.

If neither of the two scenarios noted above occur after two minutes of the encounter, begin to look nervous or distracted. If they ask you if you’re okay, go into the “Infant Sleeping Back Story” below.

Infant sleeping back story: A child in your community died in bed with their parents. You wonder why God would take a baby like that. You are unsure of whether it was God’s decision or another reason for why the baby died. You have heard that they don’t sleep with their babies in the U.S. but you know your baby would be lonely and feel cold if he slept alone and you don’t know what to do. You think you might have a pack-n-play that was donated to you, but you have never used it and would not know how to open it. You do not want to let your baby to sleep on his own but if the resident engages you about how you feel about co-sleeping and tries to understand you, you may be willing to implement strategies to reduce risks of co-sleeping.

Anticipated Management Mistakes

1.   Difficulty initiating the conversation about sleep: Some residents took several minutes to start asking about sleep, and focused instead on feeding patterns, even after the SP had admitted to unsafe sleep habits. This may be because the resident was hesitant to have the discussion with the SP. The SP could then ask the resident outright “Is it safe to sleep with my baby in bed with me?” and then the resident would need to respond in an empathetic but accurate manner. 

2.   Uncertainty about risk mitigation strategies: This may be a lack of education on the topic previously, and further strategies can be addressed during the debriefing period. The SP could help this conversation by providing examples of known risk mitigation strategies and ask the resident if they think it would make sleep safer. For example, she could say “Would it be safer to sleep with my baby if we slept with no blankets, sheets or pillow? If my husband slept in a different room? If we moved the mattress onto the floor, away from any walls?”

Appendix D: Judges’ scoring sheet

Team Names:                                                                                                              Judge:

**Table 6:** Judges' scoring sheet

1	Positive non-verbal communication - smiling at patient, speaking slowly	Delayed or incorrect performance of most criteria	Delayed or incorrect performance of many criteria	Delayed or complete performance of some criteria	Competent performance of most criteria	Efficient and rapid performance of all criteria
2	Clear verbal communication - Introduces self; assesses if interpreter is needed; engages in rapport building	Delayed or incorrect performance of most criteria	Delayed or incorrect performance of many criteria	Delayed or complete performance of some criteria	Competent performance of most criteria	Efficient and rapid performance of all criteria
3	Probes on co-sleeping practices - Asks about current sleeping environment (including number of individuals in bed), demonstrates awareness of safe sleep recommendations	Delayed or incorrect performance of most criteria	Delayed or incorrect performance of many criteria	Delayed or complete performance of some criteria	Competent performance of most criteria	Efficient and rapid performance of all criteria
4	Explores mother’s beliefs regarding co-sleeping - Asks about how the story made mother feel, empathize with mother, asks about mother’s thoughts (and/or community’s stance) on co-sleeping in non-threatening or non-accusatory manner	Delayed or incorrect performance of most criteria	Delayed or incorrect performance of many criteria	Delayed or complete performance of some criteria	Competent performance of most criteria	Efficient and rapid performance of all criteria
5	Engages in supportive interactions around the topic of co-sleeping - Acknowledges importance of mother’s knowledge/feelings about child, expresses shared goals for child	Delayed or incorrect performance of most criteria	Delayed or incorrect performance of many criteria	Delayed or complete performance of some criteria	Competent performance of most criteria	Efficient and rapid performance of all criteria
6	Additional Comments:					
